# Understanding collective regularity in human mobility as a familiar stranger phenomenon

**DOI:** 10.1038/s41598-021-98475-x

**Published:** 2021-09-30

**Authors:** Yan Leng, Dominiquo Santistevan, Alex Pentland

**Affiliations:** 1 McCombs School of Business, The University of Texas at Austin, Austin, 78712 USA; 2 Department of Sociology, University of Chicago, Chicago, 60637 USA; 3 Media Lab, MIT, Cambridge, 02139 USA

**Keywords:** Computational science, Complex networks

## Abstract

Beyond the physical structures that contain daily routines, urban city dwellers repeatedly encounter strangers that similarly shape their environments. Familiar strangers are neither formal acquaintances nor completely anonymous faces in daily urban life. Due to data limitations, there is a lack of research focused on uncovering the structure of the “Familiar Stranger” phenomenon at a large scale while simultaneously investigating the social relationships between such strangers. Using countrywide mobile phone records from Andorra, we empirically show the existence of such a phenomenon as well as details concerning these strangers’ relative social relations. To understand the social and spatial components of familiar strangers more deeply, we study the temporal regularity and spatial structure of collective urban mobility to shed light on the mechanisms that guide these interactions. Furthermore, we explore the relationship between social distances and the number of encounters to show that more significant physical encounters correspond to a shorter social distance. Understanding these social and physical networks has essential implications for epidemics spreading, urban planning, and information diffusion.

## Main

“Familiar Stranger” is a unique and pervasive urban social phenomenon because, as the name suggests, it is defined by a relationship between two people that is neither completely anonymous nor wholly familiar. Familiar Strangers are those people we encounter during regular activities in daily life but with whom we never formally interact^1,2^. This latent social network can play a significant role in information diffusion^3,4^, behavior synchronization^5^, and the transmission of infectious diseases^6–8^. Though there is no direct social interaction, individuals learn from and are affected by each other’s decisions within a shared physical space. Moreover, epidemics expand through contact that occurs in these physical spaces. Therefore the awareness of the interactions between non-friends is crucial for understanding infectious disease diffusion and designing control mechanisms.

With the abundance of behavioral data, there is a growing interest in understanding the regularity and predictability of human mobility at an unprecedented spatial scale and granularity^9–12^. Interestingly, such regularity may produce the “familiar stranger” phenomenon and can be studied directly with additional complementary social network information. Many have experiences of repeatedly identifying another individual visually without interacting. Despite the lack of interaction, such regularity is, by definition, the central condition of the urban-wide familiar stranger phenomenon and has been supported by empirical evidence in the literature. The concept of “Familiar Stranger” was first articulated by Stanley Milgram in 1972 with an experiment on a bus platform. According to his definition, the three characteristics of a Familiar Stranger are (1) observation, (2) repetition, and (3) lack of interaction. Milgram conducted a small-scale experiment by asking travelers to recognize “strangers” they could recall from their own commutes^1^. In 2013, Sun et al.^13^ uncovered the encounter mechanisms of three million public transit users in Singapore to capture the in-vehicle encounter patterns using transit smart-card data. Zhou et al. formally defined familiar strangers in the big data era and operationalized the proposed concept using the five-day smart card data and mobile location data in Beijing^14^. Riascos used data from location-based social network Foursquare and observed the existence of such encounter networks in both New York and Tokyo^15^. However, neither of the data used in these studies contains information indicating existing social interactions beyond these encounters, leaving it possible that the physical encounters are indeed between socially connected individuals who coordinated their encounters.

To capture the physical encounter network at a countrywide scale and establish that the encounters are not between actual “friends,” i.e. non-strangers, we use countrywide mobile phone data in Andorra. The data enables us to identify the physical encounters which do not share a direct mobile phone exchange, thus, by our definition, making them relative strangers as opposed to friends. With reference to Milgram’s definition above, the analogy for our data is as follows: (1) the mutual “observations” criterion is accounted for by identifying two users on the same tower within the same 15-min window, (2) “repetition” by finding multiple encounters between the same two users, and (3) “lack of interaction” through the absence of a shared call or short message service (SMS). The objective is to study whether such familiar strangers exist on a large scale and identify the underlying patterns that may help describe such a phenomenon in more detail. The call detail record (CDR) simultaneously captures two networks: physical encounter networks (mobility network) and phone communication networks (social network).

We approach the concept of “social network” cautiously and with the understanding that the exchanges recorded over the telecommunications network is only a proxy for social relationships that may exist beyond the scope of our data. For example, the relationship between colleagues in which mobile phone numbers are never exchanged yet there is genuine social interaction will not be visible in our network. Having said that, we also believe that CDR networks provide some semblance of social-relational insight beyond that which can be inferred by only using CDR as mobility information. Along similar lines, we also acknowledge limitations in the mobility component of the data. Sampling biases have been noted in CDR studies^16^, but despite these limitations, our data has recorded substantial evidence of encounters between strangers.

Using the countrywide mobile phone data, we observe the existence of the familiar stranger phenomenon in an urban environment. A series of papers have confirmed the regularity and predictability in human mobility^17,18^, and we show that the widely-observed rhythms in human mobility^17^, both temporally and spatially, may explain such a phenomenon. We further build a gravity model, widely used in the literature with various applications, to describe the occurrences of physical encounters concerning the location of the encounter. Lastly, we investigate the relationship between physical co-occurrences and the proximity in social networks. We show that the number of physical co-occurrences predicts shorter social distances on phone communication networks.

## Results

There is ample empirical evidence showing that human mobility follows a high degree of temporal and spatial regularity^17^. This evidence might suggest that physical repeated encounters do not happen by chance alone. Instead, collective regularity of human behavior results in physical co-occurrences^1,13,19^. We mainly focus on the temporal rhythms and spatial structure of two consecutive encounters of familiar stranger pairs to uncover the mechanisms that underlie the encounters as well as the latent social relationships undergirding the encounters between individuals.

### Temporal regularity

Daily routines and habitual action can and, according to our data, usually does follow specific temporal patterns. To explore the routine mobile action that results in physical encounters at a national scale, we create an encounter network based on mobility behavior across an entire month and measure the inter-event time $\Delta t$ between consecutive encounters for each user pair, specifically focusing on those pairs without a previous history of direct social contact, or what we refer to as familiar strangers.

**Figure 1:**
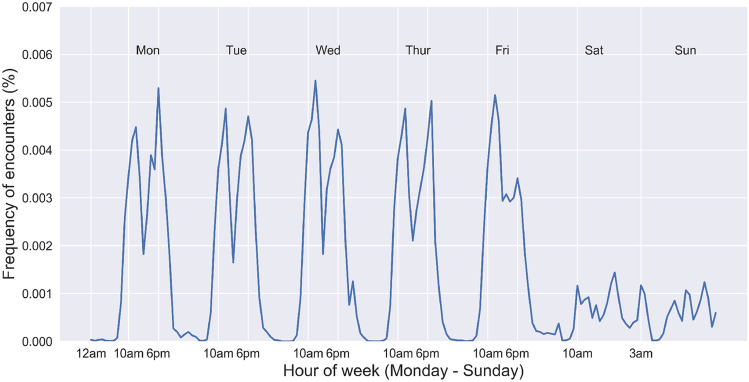
Temporal distribution of physical encounters. The x-axis corresponds to the hour within the week, and the corresponding day of the week is labeled on top of the figure. The y-axis is the frequencies. We only show one week of activity here to emphasize detail in patterns, but similar patterns are visible for all weeks of the month.

We begin by analyzing the temporal distribution of all encounters with the objective of confirming the existence of temporal regularity in such encounters, which we identify as a precondition for the greater phenomenon. It is through consistency that, as Milgrim explains, faces become a part of our environment. We first extract the time of day when an encounter occurs, which can be seen in Fig. 1. As we can see, prominent spikes exist between working hours on weekdays, from 9 a.m. to 12 p.m. and 5 p.m.–7 p.m. The patterns over the weekend are different from those over the weekdays. Specifically, there is a slight shift—the physical encounter happens from 10 a.m. to 11 a.m. and from 3 p.m. to 6 p.m. Interestingly, we also observe a small peak at 3 a.m. on Sunday, capturing a unique nightlife pattern during the weekend. This observation shows the existence of temporal regularity in human mobility, which is a strong indicator of the larger phenomenon at hand. It would appear that individuals commute to work and back home with a repeated and regular pattern making them more likely to encounter familiar strangers during those hours, while weekend behavior can be described as less rigidly scheduled as encounters are spread across the days resulting in lower peaks.

**Figure 2:**
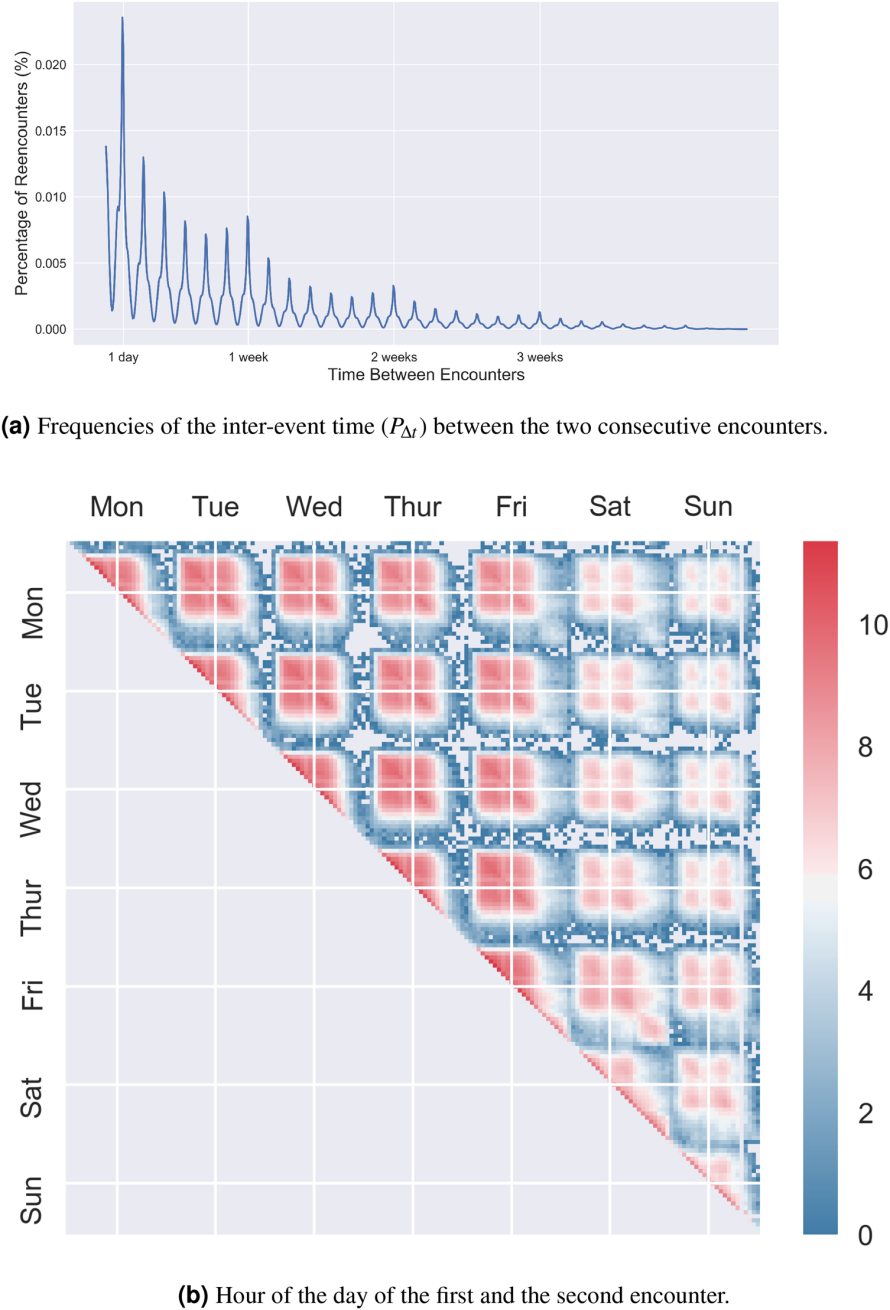
Temporal regularity. (**a**) Frequencies of the inter-event time ($P_{\Delta t}$) between the two consecutive encounters. The x-axis corresponds to the number of hours between the two repeated encounters of a pair of familiar strangers. The y-axis is the number of encounter pairs. (**b**) Hour of the day of the first and the second encounter. The y-axis and the x-axis correspond to the time of the day of the first and the second encounter. The color scale represents the frequencies of the encounters with the corresponding count ascending from blue to red.

Figure 2a presents the time between two consecutive encounters. There exist higher peaks for every 24 h and another set of lower peaks for $24 \cdot d \pm 6$ h, where *d* is the number of days after the first encounter. Further, there is another relative peak at $24 \cdot d \cdot w$ h after the initial encounter, where *w* is the number of weeks since the first encounter. This pattern indicates that individuals are likely to encounter familiar strangers on the same hour of a day within the next *d* days or *w* weeks, again highlighting the temporal regularity of human mobility behavior.

Moreover, we analyze relative encounter times positioned within the frame of a week. As shown in Fig. 2b, we observe two distinct patterns of physical encounters: weekday encounters and weekend encounters. Our data shows that individuals who encounter during weekdays are less likely to encounter each other during weekends and vice versa. Further, morning encounters are more likely to encounter again during morning time, which explains the 24-h peak presented in Fig.  2a. Both empirical results highlight that there exists collective temporal regularity in daily human routines, which further supports the larger repeated physical encounters.

### Spatial structure of physical encounters

**Figure 3:**
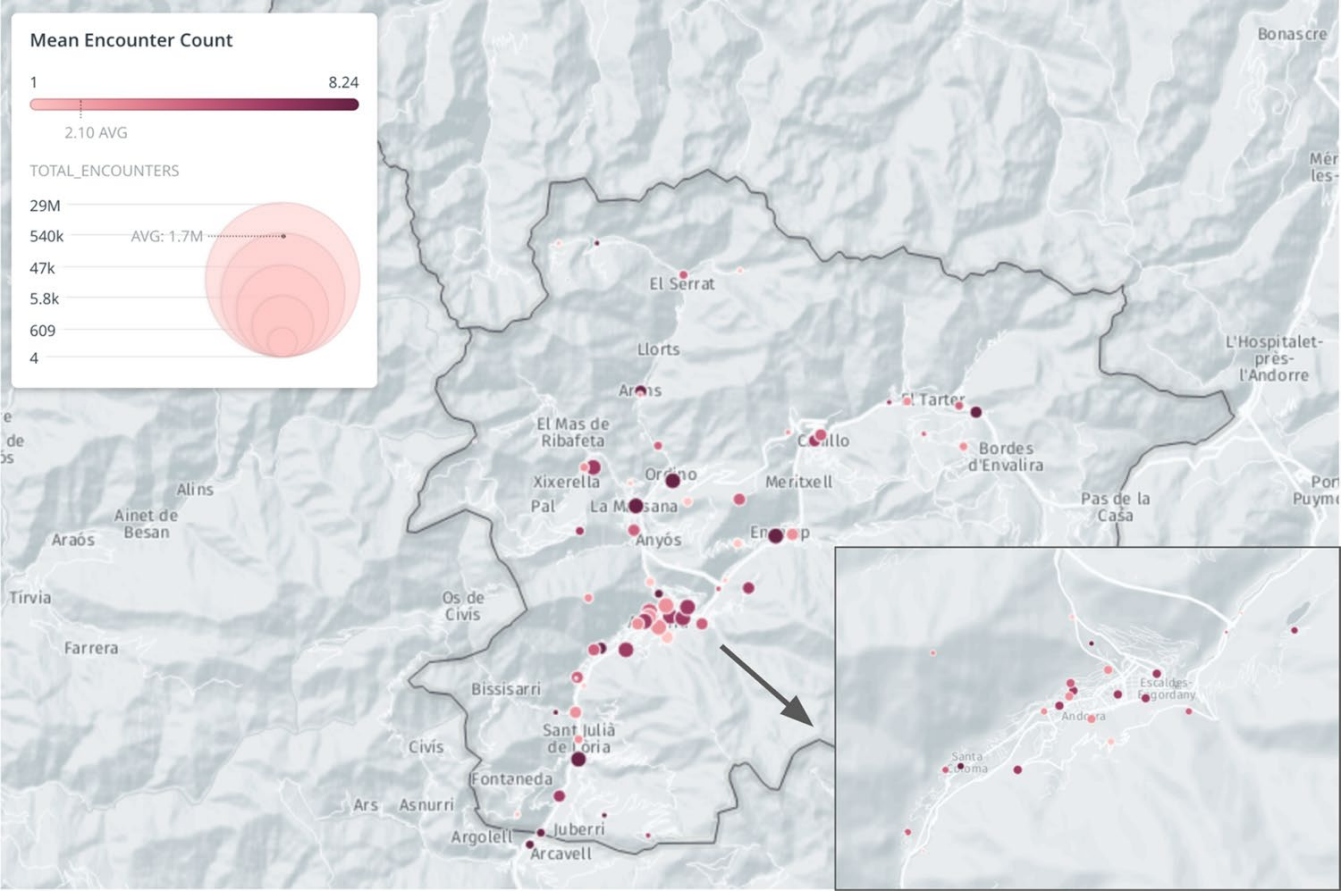
Spatial distributions of encounters. Each node on the map corresponds to one cell tower. The size of the node is proportional to the total number of encounters. Darker red corresponds to more encounters for each encounter pair. This figure is generated using CartoDB (https://carto.com).

We analyze the spatial distribution of encounters of familiar strangers aggregated at the cell tower level. Points of Interest (POIs) were provided to us by the Andorran government, which essentially provides a categorization of possible activities that take place around each observed tower. Due to the varying nature of the POI categories and corresponding tower areas, the number of total encounter pairs, as well as the frequency of encounters for each familiar stranger pair with respect to these towers, vary similarly. As shown in Fig. 3, there exist some towers that are not as popular with encounters, yet they have primarily repeated encounters between pairs of familiar strangers. This observation highlights the difference between encounter pairs with one-time encounters and those with multiple encounters. It shows that people perform regular daily activities at specific cell towers, and therefore the average number of encounters between each pair of familiar strangers is higher. By our definition, if the number of encounters between a pair of individuals is only one, they are not familiar strangers but merely faces in the crowd that have not yet become familiar.

**Figure 4:**
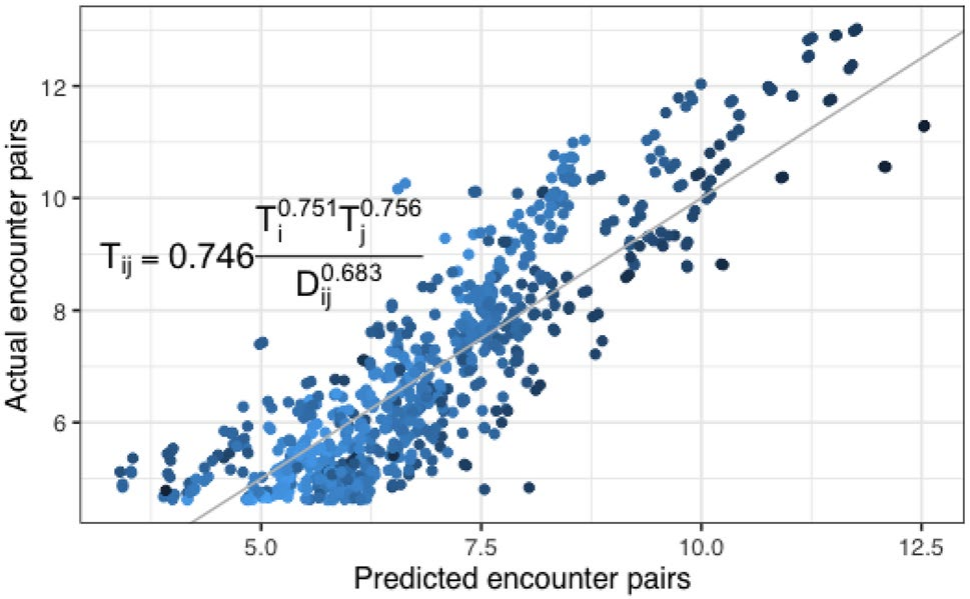
Gravity model of repeated encounters. The x-axis and y-axis correspond to the predicted and observed re-encounter pairs. The grey diagonal line represents perfect predictions.

We use a gravity model to understand how the quantity of repeated encounters can be predicted by the spatial distances and popularity of two potential encounter locations. We adopt the simplest functional form of the gravity model,

1 $$\begin{aligned} T_{ij} = C \frac{N_i^\alpha N_j^\beta }{D_{ij}^\gamma }, \end{aligned}$$

where $T_{ij}$ is the amount of encounters and re-encounter flow between two geographical locations *i* and *j*. $D_{ij}$ is the distance between geographical region *i* and *j*. $N_i$ and $N_j$ are the number of physical encounters at region *i* and *j* respectively. We demonstrate the fit of the gravity model, the relationships between encounter and re-encounter flows, and the variables of interests in Fig. 4. *C*, $\alpha$, $\beta$ and $\gamma$ are the exponential parameters to estimate. We apply a logarithmic transformation and fit the parameters with a linear regression. Our estimates show that $C = 0.746$, $\alpha = 0.751, \beta = 0.756, \gamma = 0.683$. $\gamma$ being positive indicates that the closer the two physical encounter locations, the more likely the encounter happens repeatedly. A positive $\alpha$ and $\beta$ indicates that the more popular the encounter locations, the more frequent the repeated encounters. The effects of both are similar.

### Spatial pattern of re-encounter: points of Interests

**Figure 5:**
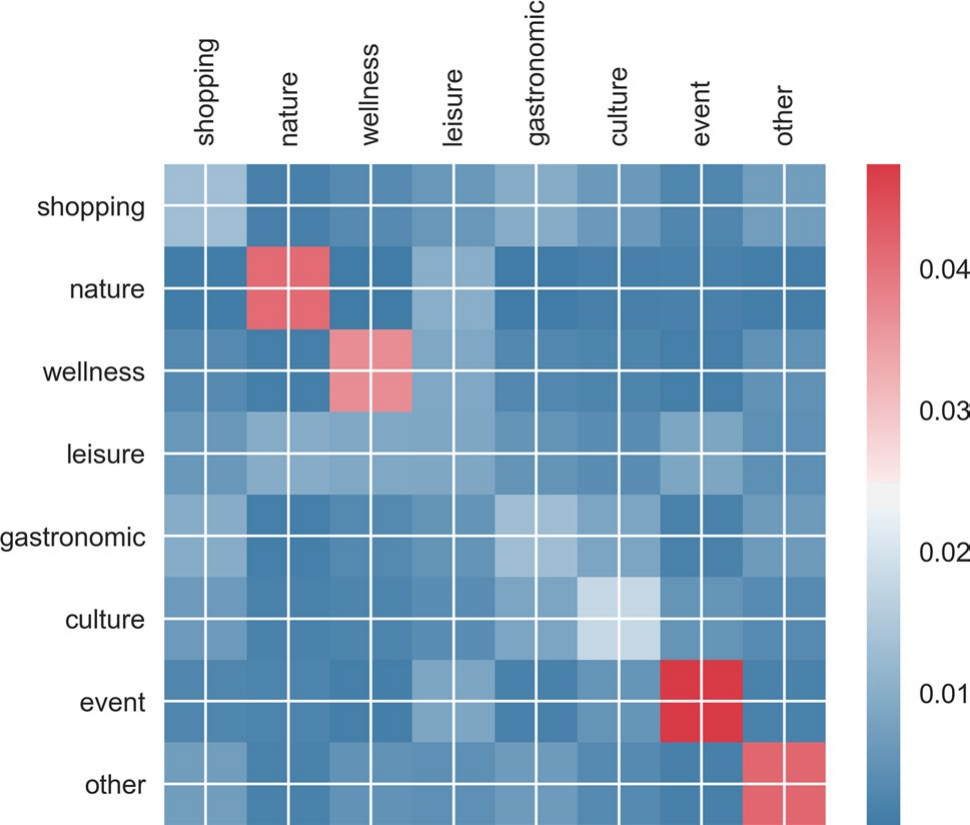
Normalized transition probabilities from one to another type of POIs. The x-axis and y-axis correspond to the type of POI of the first and the second encounter. From blue to red correspond to infrequent to frequent pairs of POIs.

A Point of Interest is a specific location that someone may find useful or attractive, such as a skiing resort or a museum. This term widely appears in geographic information systems. The category of POI potentially indicates the purpose and/or activity of a given trip within transportation studies^20^. To associate cell towers with potential trip purposes^22,23^, we manually assign eight types of POIs to cell towers including shopping, nature, wellness, leisure, gastronomic, outline, event, and others, if these POIs fall within the cell towers' coverage by Voronoi tessellation^21^. By analyzing the POIs surrounding the two following locations in Fig. 5, we observe that individuals are more likely to encounter one another at the same type of locations, specifically in nature, wellness, event, and non-touristic places. This pattern indicates that people who share the same interests are likely to encounter each other at another type of location. This pattern further shows that homophily exists in not only social network^24^ but also in this underlying familiar-stranger relationship.

## Familiar strangers in social network

**Figure 6:**
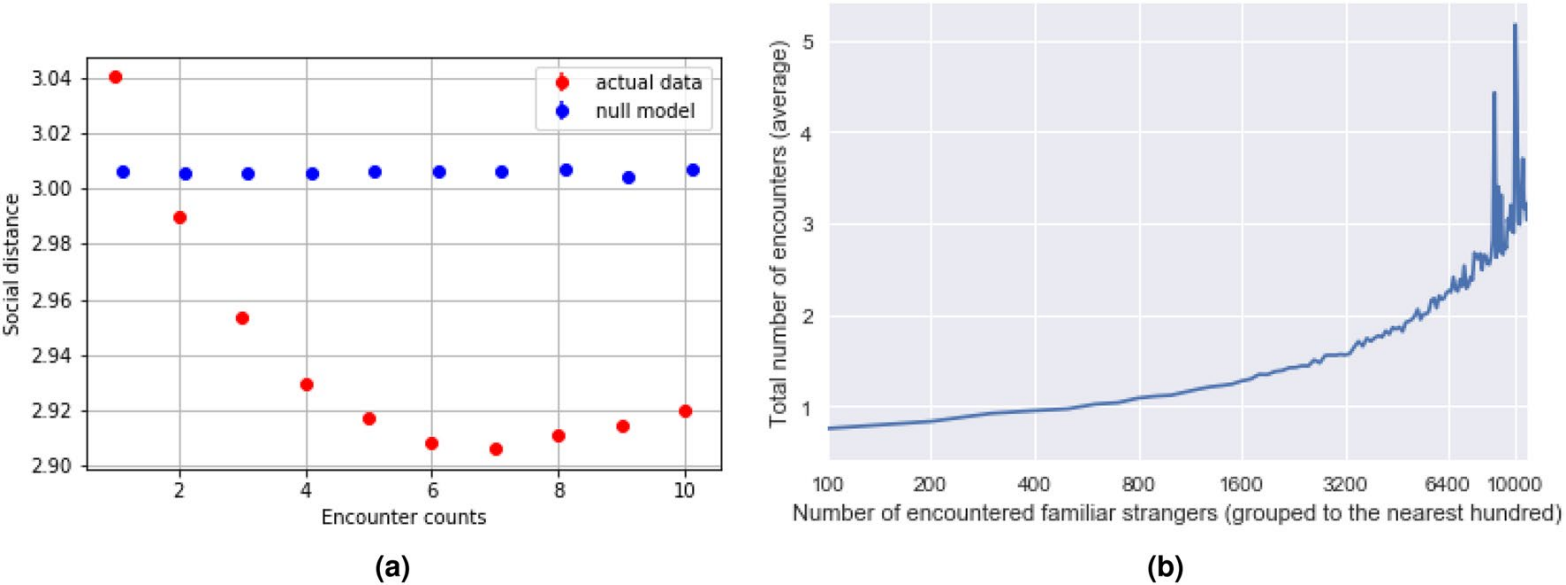
(**a**) Relationship between the number of encounters and social distances in social networks of familiar-stranger pairs. In the left panel, the x-axis is the number of encounters for each familiar-stranger pair. The y-axis is the average social distance of the corresponding number of encounters. The red and blue points correspond to the real and the shuffled data. (**b**) In the right panel: the x-axis shows the number of familiar strangers and has been grouped to the nearest hundred and shown on a log scale to highlight the increasing trend. The top 1% of encountered counts have been removed in an attempt to remove anomalous users.

Next, we study the social distances between pairs of familiar strangers. Crandall et al. find that the probability of a social tie increases sharply as the number of occurrences increases^25^. Toole et al. show that users’ ego networks with immediate connections share similar mobility behavior^26^. Aside from the observed relationship between mobility behavior and the probability of tie formation (or strength), we are specifically interested in the social distances between “familiar strangers”. As shown in Fig. 6a, social distance correlates negatively with the number of encounters. In other words, the more significant number of times each familiar-stranger pair encounters one another, the shorter the graph distance between them on the phone communication network. As a null model, we shuffled the social distance among the familiar strangers such that the total distribution of social distance is the same as the original data. We see that the pattern disappears after the shuffling process. In Fig. 6b, we extend this analysis to the individual level. As the number of encountered familiar strangers increases, the total number of encounters also grows, demonstrating the reinforcing effects of collective regular behavior.

## Discussion

Milgram has formally identified the phenomenon of a familiar stranger via a small-scale experiment^1^. Due to the data limitations, there have been limited studies focusing on describing the phenomenon at a wider scale, investigating the social relationships between familiar strangers, and understanding the development of the relationships through time. With large-scale mobile phone records, we empirically show the existence of familiar strangers in Andorra. By analyzing the temporal and spatial characteristics of the encounters, we uncover the underlying mechanisms, especially collective temporal regularity and spatial structure that trigger the phenomenon. In the end, we explore the relationship between social distances along with social networks and the number of encounters in a mobility network. We show that a more substantial amount of encounters predicts shorter social distances on social networks, and the majority of re-encounters occur periodically, which makes salient exactly when a potential “weak tie” is established^27^. Though we have established a relationship between the number of encounters and social distance, there remains work to describe the different contexts from which a “stranger” becomes a friend by establishing a clear link. For example, this would allow researchers to empirically test Milgram’s hypothesis that familiar strangers are likely to formally engage if they mutually identify each other in a location other than their standard meeting point. For Milgram, it is actually less likely than familiar strangers will spontaneously interact in the usual location because that relationship has been solidified in its passivity, so it is only in a new setting that the interaction barrier can be broken. Also, not all familiar stranger encounters are the same, so the characteristics and friendship development of different types, e.g., commute strangers versus familiar strangers seen repeatedly on a weekend night out, may be another next step towards understanding the social world of the, once thought, anonymous urban individual. The understanding of the physical encounter network could have significant implications for epidemics prevention and the facilitation of information transmission.

Understanding the familiar stranger phenomenon has many social applications, such as epidemics control, urban planning, and marketing. First, the familiar strangers encounter patterns shed light on epidemic development and may help design vaccination and organization strategies to mitigate spread. For example, in epidemics, we can prioritize the blocking of locations with a larger ratio of physical encounters to the number of re-encounters to reduce the potential large-scale infections since these locations will be the site of more “random” encounters, reinforcing naturally grouping partitions. Note that places with many physical encounters may differ from locations with the most substantial flows. The reason is that the former are places where people visit in the same period, while the latter may have distributed flows across the period. Besides, Christakis et al. developed a “social sensor” strategy to monitor individuals with a large number of friends^28^. We believe that monitoring individuals with many physical encounters can further improve the “social sensor” strategy. Moreover, with more data on human mobility and social interactions, we can study how spatial structures and city design influence familiar strangers’ interaction patterns. For urban planners, insights on familiar strangers help provide implications on policy design and transportation system operations. For example, Sun et al. demonstrate that the spatial-temporal regularity in familiar strangers can be utilized to detect anomalies in individual travel behaviors^29^, which can be useful for urban emergency management. In addition, urban planners can use these insights to design strategies and infrastructures or organize local events to encourage physical encounters. It will be interesting to understand whether ride-sharing systems can serve as an effective mechanism for bonding familiar strangers, which can provide insights into the matching algorithms on ride-sharing platforms. Lastly, marketing companies can leverage hidden social influence among familiar strangers to advertise for new products^4^. For example, they can target individuals who encounter a larger number of familiar strangers with promotional souvenirs in Andorra. If these individuals adopt these souvenirs and carry them around, these individuals can help to spread information.

However, there are still questions about the details of when and how links are formed between “strangers.” There are different forms of social relationships—some strong, regularly occurring, with emotional intensity and intimacy, while others might be considered “weak,” given the relationship’s existence but lack of intensity—which is crucial for understanding the connection of potentially disparate communities^27^. On the one hand, there exists plenty of empirical evidence highlighting the role of physical encounter networks in the development of group norms and behavior. On the other hand, it is not clear how the “familiar stranger” can transition into more sustained friendships. In the age of the metropolis and the subsequent studies of early sociological thinkers, urban anonymity has been a point of both lamentation and curiosity because of the apparent resulting freedom for the individual; however, the familiar stranger exists between them these worlds^30^. In a study of an apartment complex in New York, neighbors made little to no effort to formally engage in “neighborly” interactions beyond an occasional “hello.” Yet, most tenants confidently identified “self-other” characteristics and developed opinions about their repeatedly encountered neighbors and even spoke about those judgments with other neighbors^31^. This result can signal that, despite little to no formal engagement, some social, behavioral constraints may still develop; thus, the anonymous, isolated, urban life maybe less anonymous, and therefore less “free” than originally assumed. As shown by^13^, the connections between familiar strangers grow stronger over time. Mobility similarities indicate strong similarities in preferences and socio-demographics. Hence, we might believe the “Familiar Stranger”-driven relationships are more stable. Further studies are required to test this hypothesis using longitudinal data. Our analysis is performed in the country of Andorra, and we leave the generalization to other countries for future studies.

## Data and materials

The social and mobility networks used in this study were created from anonymized mobile phone data collected in Andorra, a European Country, from April 1, 2016 through July 31, 2016. Call Detail Records (CDR) is an opportunistic, large-scale, longitudinal data source that enabled us to observe mobility behaviors at both individual and aggregate levels. The high penetration rate of mobile phones together with their frequency of use, make CDR a useful signal for observing urban-wide human behaviors. While CDR data alone does not create a complete picture of individual or group tendencies with respect to socializing and mobility, the foundational assumption of our study was that CDR’s sampling of social behavior did provide a proximate heuristic for inferring larger patterns, especially when the data analyzed is at the scale of an entire country.

CDR data was originally gathered and stored for telecommunication purposes, such as billing and troubleshooting, however, it also implicitly contains both mobility histories and the social networks derived from phone communication patterns between users. Specifically, for every mobile network connection made, whether via calls, SMS, or data request, the information from that exchange with the telecommunications network is stored. This information includes an anonymized identifier for the caller and receiver, the cell tower used in connecting, and the start and end times of the connection. With complementary information on the location of each tower, we are able to infer approximately where a user was located when the connection was made. Further, with the approximate inferred location of multiple callers and the time at which the connection was made, we were able to observe repeated co-location of callers and thus observed familiar strangers. While real events are much more fluid, for example the service area of the towers may vary or a person may move between towers during a connection, i.e. signal migration, we only consider the first tower to which a user connected in the 15-min window as a discrete marker for location at a given time. The service area is provided by the mobile carrier—in general, the service area may differ across rural versus urban areas. In this study, we created two separate but complementary structures from the same data - the social networks and another to identify the encounter location pairs.

We first created a communication network, or social network, in which each user was a vertex and an edge existed if there was a direct sender-receiver connection between users, by either call or SMS. The social network was made from all calls and SMS entries from the beginning of April through July 2016. There were a total of 2,782,069 users in our network sample. The degrees, or the number of neighbors, of the vertices in the initial social network graph, ranged from 1 to 31,602. Given that the 99.99th percentile of degree values of the social graph is 468, we remove all vertices with a degree value above the 468 neighbors threshold in the interest of removing highly anomalous users, leaving 2,781,791 users in our social graph. The “social distance” value itself is derived by identifying the shortest path between two users within the final social graph.

Before identifying encounters, we proceeded to create filter criteria for the raw data with the intention of identifying which users are regular Andorran urbanites and which users are not. Andorra is positioned between France and Spain, and a known tourist destination, and as such, many users do not actually live in Andorra. We removed all users with a foreign identification code in the CDR as well as those who did not have at least one incoming and one outgoing call or text. We also did not consider all towers in the region of El Pas de la Casa, a small ski resort town bordering France. This left us with 54,758, which is plausible, given the country’s total population is estimated to be 77,000 in 2019^32^. To identify the encounters themselves, we partition the total data by tower and slide a 15-minute window across the ordered records, storing every pair of co-occurring users. Each unique pair of users from all towers were then aggregated, which allowed us to identify re-encounters. We then identified the shortest path length^33^ between each pair of users in our social network. In total, we identified 51,849,343 unique encounter pairs for a total of 140,665,452 instances of an encounter. Finally, we made a distinction between friends, strangers, and familiar strangers. A pair of users were considered friends if they shared an edge with strangers being the complement to this set, however a pair of users is only a “familiar stranger” if they are strangers with more than one encounter.

## Data Availability

The datasets generated during and/or analysed during the current study are not publicly available subject to data use agreements. The processed data to generate all the figures are available from the corresponding author on reasonable request.
